# Effects of Anthropogenic Disturbance and Climate on Patterns of Bat Fly Parasitism

**DOI:** 10.1371/journal.pone.0041487

**Published:** 2012-07-19

**Authors:** Shai Pilosof, Carl W. Dick, Carmi Korine, Bruce D. Patterson, Boris R. Krasnov

**Affiliations:** 1 Mitrani Department of Desert Ecology, Swiss Institute for Dryland Environmental and Energy Research, Jacob Blaustein Institutes for Desert Research, Albert Katz International School for Desert Studies, Ben-Gurion University of the Negev, Beer-Sheva, Israel; 2 Department of Biology, Western Kentucky University, Bowling Green, Kentucky, United States of America; 3 Department of Zoology, Field Museum of Natural History, Chicago, Illinois, United States of America; University of Regina, Canada

## Abstract

Environmental conditions, including anthropogenic disturbance, can significantly alter host and parasite communities. Yet, our current knowledge is based mainly on endoparasites, while ectoparasites remain little studied. We studied the indirect effects of anthropogenic disturbance (human population density) and climate (temperature, precipitation and elevation) on abundance of highly host-specific bat flies in four Neotropical bat species across 43 localities in Venezuela. We formulated a set of 11 *a priori* hypotheses that included a combination of the two effectors and host species. Statistically, each of these hypotheses was represented by a zero-inflated negative binomial mixture model, allowing us to control for excess zeros in the data. The best model was selected using Akaike's information criteria. Fly abundance was affected by anthropogenic disturbance in *Artibeus planirostris*, *Carollia perspicillata* and *Pteronotus parnellii*, but not *Desmodus rotundus*. Climate affected fly abundance in all bat species, suggesting mediation of these effects via the host or by direct effects on flies. We conclude that human disturbance may play a role in shaping bat-bat fly interactions. Different processes could determine fly abundance in the different bat species.

## Introduction

All organisms, including parasites, are influenced by the environment in which they occur. Although parasites are enormously diverse and ubiquitous in distribution, our understanding of environmental effects on host-parasite dynamics in wildlife is limited mainly to aquatic environments (e.g., [1], [2]). Recent research in terrestrial habitats has mainly investigated the extent of anthropogenic disturbance on endoparasites (e.g., [3], [4]), whereas ectoparasites have been largely neglected (but see [5]). It is imperative to investigate the effects of the environment (e.g., climate and anthropogenic disturbance) on ectoparasites in terrestrial habitats because ectoparasites exert important selective pressures on the evolution of their hosts [6] by affecting host fitness and population dynamics [6], [7].

One fundamental metric of parasite pressure is parasite abundance (number of parasites infecting an individual host). Environmental effects on parasite abundance can be direct, operating on the parasites themselves. For example, parasite abundance increases where local conditions promote parasite development [8] but decreases where environmental contaminants are harmful for the parasite [9]. Environmental effects can also be indirect, affecting parasite abundance through their effect on the host. For instance, parasite abundance increases where conditions act to increase host density [10] or suppress host immunity [11]. However, the processes that determine parasite transmission and establishment are governed by multiple factors that operate simultaneously and whose effects are usually difficult to separate [12].

Anthropogenic disturbance may influence parasite abundance via effects on local host species richness and abundance (e.g., the “dilution” effect [5], [13], [14]). However, the dilution effect is expected to pertain only to generalist parasites because they select from a range of host species, while specialist parasites are limited in their choice among host species [13]. Therefore, in highly host-specific parasites, environmental effects on parasite abundance are not expected to be a consequence of changes in local host species richness but rather a result of changes in the local densities of the hosts themselves. In addition, the distribution of highly host-specific parasites should follow that of their hosts. Therefore, variation in parasite abundance can be mainly attributed to indirect environmental effects on hosts. Yet, abundance of ectoparasites, which are also exposed to the off-host environment, can also be influenced directly by external factors such as temperature and precipitation [15], [16].

We used Neotropical bats and their parasitic bat flies (Diptera: Streblidae) to investigate the effects of climate and disturbance on fly abundance. Neotropical bats occupy both natural (undisturbed) and human-disturbed habitats and some species have a vast distribution, spanning numerous geographical regions [17]. Bat flies are highly host-specific, with most species parasitizing one or two host species [18]. Flies are obligate ectoparasites of bats and have an indirect transmission cycle. Female bat flies leave a host to deposit larvae in the bat's roost. Larvae immediately pupate and undergo metamorphosis over ca. three weeks. Females and, later, the newly eclosed flies, must then locate an appropriate host species, often in roosts shared by multiple bat species [18].

The association between Neotropical bats and bat flies is strongly influenced by the roosting habits of the host [19], [20]. Durable roosts accommodate more bat individuals, and do so more predictably, increasing the likelihood of bats being present for colonization by newly eclosed flies. Therefore, bats using exposed, ephemeral roosts such as leaves typically have lower fly abundance than those that roost in more protected, durable roosts such as caves or tree holes. Roost use, in turn, can be affected by anthropogenic activity [21], [22]. For example, foliage- or tree hole-roosting bats may encounter fewer suitable roosts in fragmented forests with fewer old, hollow trees, forcing them to congregate in the remaining roosts, thus promoting parasite transmission [3]. Alternatively, bats can take advantage of human-made structures and use them as roosting sites [23]. Apart from the effects of roost availability on local host abundance, variation in local climatic conditions can further affect variation in local host abundances through effects on food availability [24], [25].

Our goal was to analyze the effects of climate and anthropogenic disturbance on fly abundance. We hypothesized that fly abundance would be indirectly influenced by climatic conditions and anthropogenic disturbance through their effect on local bat abundances or directly through climatic effects on flies themselves. Because it is difficult to predict the magnitude and direction of climatic effects on fly abundance, we did not generate specific predictions. However, this is not the case with anthropogenic disturbance such as clear-cutting that affects roost availability. We therefore predicted that fly abundance of tree-roosting bats will increase in more disturbed areas due to increased bat aggregations in remaining available roosts.

## Methods

### The dataset

The largest-ever coordinated survey of Neotropical mammals and their ectoparasites was carried out in Venezuela by researchers from several organizations between 1965–1968 [26]. Over 30,000 mammals and their ectoparasites were sampled across Venezuela in the framework of the Smithsonian Venezuelan Project (SVP). Of these, 24,138 individual bats (Chiroptera) were surveyed for streblid bat flies (Diptera: Streblidae). Bat flies were collected from ether-fumigated bats and kept in 70% ethanol. To avoid cross-host contamination of ectoparasites, bats were held in individual paper bags until processing. Details on field sampling and bat and ectoparasite identification are available in Wenzel [27]. Each infracommunity (assemblage of flies recovered from an individual bat) received the unique SVP number of its host. Bat and fly sampling resulted in a comprehensive dataset of bat-fly associations. Each record in this dataset represents a fly species collected from an individual bat host such that multiple records appear when 2 or more fly species were collected from the same individual bat. Additional information for each record includes the number of flies of a particular species, and the name of the sampling locality.

Geographical information on sampling localities is taken from Handley [26]. In total, bats were captured at 43 of the primary localities mentioned in Handley [26] gazetteer (Supporting Information, Table S1 and Fig. S1). Some of these primary localities had secondary localities (i.e., sub-localities referenced to the primary localities without further description or specific coordinates) associated with them. The gazetteer contains detailed information including a description of the local habitat, geographical coordinates and a list of SVP numbers collected. We complemented the bat-fly dataset with geographical information (name and coordinates of primary capture localities) by matching the SVP numbers given in the bat-fly dataset with those of the gazetteer using an *ad hoc* computer script (written in R by D. Toubiana and S. Pilosof) to minimize potential errors. Coordinates and SVP numbers were available for primary localities only, so we ignored information on secondary localities. Then, we excluded all records in which a bat or fly were not identified to species. The resulting dataset contained information on 137 bat species, some not or only sporadically parasitized. We then selected the four most common and broadly distributed species that differed in roosting habits. These were *Artibeus planirostris*, *Carollia perspicillata*, *Desmodus rotundus* and *Pteronotus parnellii*. *Artibeus planirostris* roosts mainly in foliage and tree holes; *C. perspicillata* roosts mainly in tree holes, but also uses more protected roosts such as rocks, old buildings and tunnels; *D. rotundus* uses caves, tree holes, underneath bridges, but very rarely abandoned buildings; *P. parnellii* mainly uses caves, but occasionally also tunnels and old buildings [19], [28].

Because the dataset contained records of bat-fly associations that could be rare, equivocal or attributed to contamination [29], we used Dick's [29] definition of primary bat-fly associations, and included only those bat-fly associations in which a bat species harbored ≥5% of the total individuals of a given fly species. Finally, we used only primary localities where ≥10 individual bats were captured per species.

### Explanatory variables

In our analyses, we used bat species, seven climatic variables (mean annual temperature, mean annual precipitation, mean temperature of the warmest quarter, mean temperature of the coldest quarter, mean precipitation of the wettest quarter, mean precipitation of the driest quarter, and elevation; Table S1) and one variable describing anthropogenic disturbance (an estimate of human population density) as independent variables. The selected climatic variables may potentially influence bat-fly associations through indirect effects on distribution, foraging success and/or roosting habits of bats [25] or on fly development. We derived these climatic variables at each locality from Geographic Information System (GIS) layers available at WorldClim (www.worldclim.org). These climatic variables are based on data collected at meteorological stations between 1950–2000 at a ∼1 km pixel resolution and are available only as averages for these 50 years [30]. Human population density correlates positively with anthropogenic disturbance [31]. We obtained human population density estimates (HPE) for 1960 at each locality from the Latin America and Caribbean Population Distribution Database [32], [33] provided by the United Nations Environmental Programme (http://na.unep.net/siouxfalls/datasets/datalist.php). This database is a GIS layer which specifies the number of inhabitants in each grid cell (∼5 km spatial resolution), based on hundreds of surveys and sources.

Extracting climatic and anthropogenic variables at the exact coordinates of each locality from Handley [26] may entail potential problems because (1) slight errors in the provided coordinates might exist since they were derived from cartographic sources; (2) some bats were sampled at secondary localities, albeit in proximity to the primary localities; (3) bats are highly mobile and might thus be exposed to environmental conditions in areas other than their immediate site of capture. To overcome these problems, we first corrected the coordinates of the primary localities where bats were captured: When the coordinates pointed to a location at sea, we set the primary locality at the closest shore. We then calculated the mean of each variable within a 5 km radius buffer drawn around each primary locality.

Because climatic variables can be correlated with each other and reflect different scales (e.g., temperature vs. precipitation), we replaced them with the scores of principal components extracted by principal component analysis (PCA). The first two principal components explained 90% of the total variation (eigenvalues of 4.08 and 2.23, respectively; loadings of the variables related to temperature and precipitation were all positive). Factors related to temperature and elevation had a contribution of 88% to the first principal component (PC1), whereas factors related to precipitation had a contribution of 89% to the variation in the second component (PC2). We therefore had four explanatory variables, namely host species, PC1 (reflecting temperature-related variables), PC2 (reflecting precipitation-related variables), and HPE.

### Data analysis

Our aim was to test the importance of climate and anthropogenic disturbance in determining fly abundance across individual bats within bat species. Because each of these factors represents a different, *a priori* biological hypothesis, we applied the general statistical framework of model selection [34]. We used a series of generalized linear models and selected the best model using Akaike's information criterion (AIC) [34], [35] and calculated model probabilities, *w_i_* [34]. Model probabilities give a measure of the plausibility, on a percentage scale, that a certain model is the best model; this avoids arbitrary cut-off points in identifying the best model based on Δ AIC (i.e., difference in AIC between the current and best model; [34]).

AIC values and model probabilities do not provide information on the goodness-of-fit of each model (i.e., how far it is from the “full reality”; [34]). Because the best model in a set of candidate models is not necessarily a “good model”, we also calculated pseudo-R^2^ values (hereafter regarded as R^2^) following Nagelkerke [36] for each model as a measure of model fit. The value of R^2^ is based on maximum-likelihood estimations of the null model (i.e., intercept-only model) and the model in question. This gave us a second measure to evaluate the importance of each explanatory variable because the ratio of the likelihoods reflects the improvement of the model in question over the intercept (null) model. Furthermore, R^2^ values are normalized to 0≤ R^2^ ≤1, allowing for intuitive comparison among models. Nonetheless, the sensitivity of R^2^ to distinguish among models is low, whereas that of AIC is high [34]. Therefore, we regarded R^2^ as a heuristic.

Parasite count data usually contain excessive counts of zero [37], [38]. To account for the large number of zeros, we modeled numbers of flies on each host species using zero-inflated negative binomial mixture models (ZINB). Detailed discussion of the logic, mathematics, methodology and interpretation of ZINB models can be found in [39], [40], [41], [42]. For a detailed description of our statistical procedures, see Protocol S1 (Supporting Information). In brief, there are two sources of zeros in ecological data. “False zeros” are generated by, for example, observer errors or temporal absence of a species (e.g., a fly species) during a survey, while it may occur at other times. If a fly species is available but does not occur on a given host species or in a given sampling locality because this habitat is unsuitable for this species, then the zero count is a “true zero”. In a mixture model, the complete distribution of the estimated counts is represented by two separate components: a zero component modeling the probability of excess zeros and a count component accounting for the true zeros and non-zero counts [39], [41], [42]. Mixture models allow specifying factors that may affect the probability of obtaining a false zero (e.g., the probability of detecting a fly may change among host species). Alternatively, all zero counts may have the same probability of being false zeros [39], [40], [41], [42].

Our models incorporated dummy variables to account for the different levels of host species. Model parameters (i.e., the estimated model coefficients) of each dummy variable are calculated relative to a reference level which is chosen arbitrarily among the factor levels, while the coefficient of the reference level (intercept of the model) is calculated relatively to zero [43]. For continuous variables, the slope of the linear fit of a certain level is added to that of the reference level. We were interested in the difference of the count component parameters from zero. Consequently, we ran the best model four times, sequentially selecting each host species as the reference level for each run as suggested by [43]. A ZINB has a natural log link function, and thus negative parameter values do not result in negative counts (see Protocol S1).

Our model set for fly abundance included 11 models (Table 1) reflecting the effect of host species, climate (PC1 and PC2), anthropogenic disturbance (HPE) and possible combinations of these variables. Models included interactions with bat species (asking, for example, whether the effect of HPE on fly abundance depends on bat species) because different species may respond differently to environmental conditions. We limited our models to second-order interactions. In each of the 11 models, we used the host species as an explanatory variable of the zero component, hypothesizing that the probability of obtaining a false zero would be affected by host identity. In addition, our model set included a null model, a global model (i.e., a model that contains all possible parameters) and models representing the effects of each of our explanatory variables alone, including a host species model (Table 1). The null model is used for comparing a certain parameterized model to an intercept-only model (i.e., null). For instance, in a situation where a null model is better than a model that contains the explanatory variable host species, it can be concluded that host species is not an important explanatory variable in affecting the response variable. Therefore, we did not incorporate host species in the null model. We present detailed results only for the best model.

**Table 1:** Comparison of models used to test the effect of host species, climate and disturbance on fly abundance in four species of bats in the Smithsonian Venezuela Project data set.

Model ranks	Model structure	K	R^2^	AIC	Δ AIC	*w*_i_
1^a^	Host*PC1 + Host*PC2 + Host*HPE	21	0.25	17180	0	99.96
2	Host*PC1 + Host*PC2	17	0.247	17196	16	0.04
3	Host*PC2	13	0.229	17328	148	0
4	Host*PC1	13	0.225	17356	176	0
5	Host*HPE	13	0.222	17378	198	0
6^b^	Host	9	0.211	17451	271	0
7	PC1 + PC2	8	0.079	18337	1157	0
8	PC1	7	0.077	18349	1169	0
9	PC2	7	0.063	18435	1255	0
10	HPE	7	0.06	18453	1273	0
11^c^	1	3	0	18806	1626	0

Models are ranked from the most supported (best model) to the least supported according to Akaike information criteria (AIC). Δ AIC – difference in AIC between the current and best model; *w*_i_ – model probabilities. K – number of parameters in the model. Note that the number of parameters includes *k*, which is the dispersion parameter of the negative binomial distribution (see Protocol S1 for details). Host – host species; PC1 and PC2 – principal components of the seven environmental variables; HPE – Human population density estimate (see Methods for details).

a Global model; ^b^ host-species model; ^c^ Null model.

We used ArcGIS Desktop (ESRI, version 9.3) for all spatial analyses, and the R software environment [44] for all statistical analyses. We used the princomp function for PCA analysis and the zeroinfl function in the “pscl” package [40], [45] for ZINB modeling.

## Results

The mean values of the explanatory variables at the 5 km buffer are presented in Table S1 of the Supporting Information. *Artibeus planirostris* (n = 1569) was captured in 30 primary localities, *C. perspicillata* (n = 3404) in 35 localities, *D. rotundus* (n = 769) in 21 localities and *P. parnellii* (n = 345) in 10 localities.

The explanatory variable in each of models 8–10 (Table 1) explained 6–7% of the variation in fly abundance. However, the host species model (model 6, Table 1) explained 21% and was ranked the highest among models with one explanatory variable. The best model (model 1, Table 1) had a 100% probability of being the *a priori* hypothesis supported by the data. This model included the combined effects of climate, disturbance and host (Table 1).

**Figure 1:**
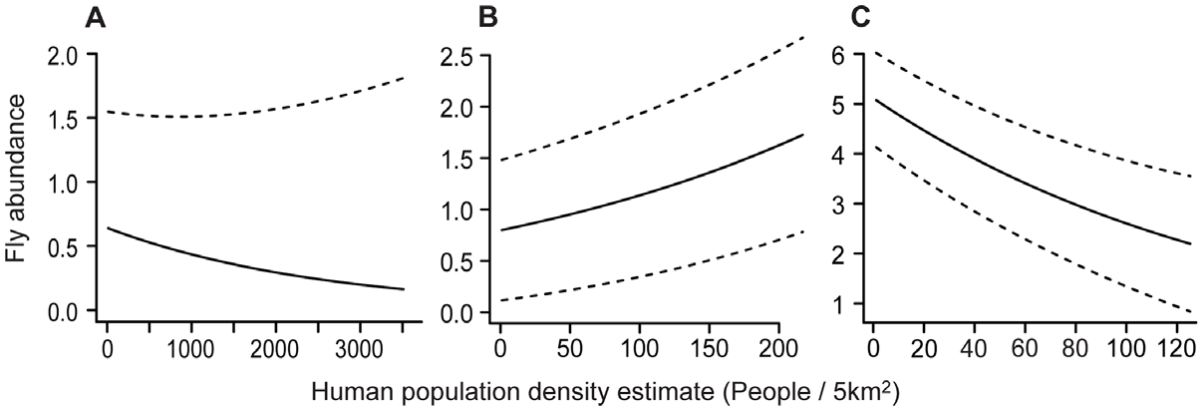
Predicted fly abundance. Fly abundance predicted across the range of human population estimate (HPE) in which (a) *Artibeus planirostris* (b) *Carollia perspicillata* and (c) *Pteronotus parnellii* occurred in the Smithsonian Venezuela Project data set. HPE was estimated as the mean number of inhabitants at a 5 km radius, as extracted from a GIS layer which specifies the number of inhabitants in ∼5 km spatial resolution (see Methods for details). Dashed lines represent the standard error of the mean and are not presented below zero.

According to the parameter estimates of the best model (model 1, Table 1), the odds for a false zero were significantly affected by host species identity. The estimated odds of observing an excess zero were 0.19 in *A. planirostris*, 0.56 in *C. perspicillata*, 0.28 in *D. rotundus* and 0.18 in *P. parnellii* (see Protocol S1 for details on calculation). Fly abundance was affected by temperature (PC1) and precipitation (PC2), although the direction and magnitude of these effects varied among species (Table 2). HPE had a significant effect on fly abundance in *A. planirostris*, *C. perspicillata* and *P. parnellii* (Table 2). To fully understand the magnitude of these effects it is fundamental to observe the values of the model parameters, because these parameters represent the intercept of each species and the slopes of the variables in question. The intercept values demonstrated that the mean values of fly abundance, taking into account the lack of any environmental effect, were significantly larger in *D. rotundus* and *P. parnellii* than in *A. planirostris* and *C. perspicillata* (on average 8 and 6 vs. 0.76 and 1.2, respectively). To demonstrate the size of the effect of HPE on fly abundance in *A. planirostris*, *C. perspicillata* and *P. parnellii*, we used the parameter values from Table 2 (see Protocol S1 for details on calculation) to predict the value of fly abundance as a function of HPE, within the range tested by the model (Fig. 1). It is evident from Fig. 1 that within the range of HPE in which each species occurs, average fly abundance declined by 25% in *A. planirostris* (from 0.64 to 0.16), increased by 46% in *C. perspicillata* (from 0.8 to 1.72), and decreased by 43% in *P. parnellii* (from 5.1 to 2.2).

**Table 2:** Parameter values for the best model (model 1 in Table 1) describing the effects of disturbance and climate on fly abundance per bat host species.

	Parameter	SE	z	P value
COUNT COMPONENT				
Aj (Intercept)	−0.271	0.082	−3.29	**0.001**
Cp (Intercept)	0.217	0.061	3.55	**<0.001**
Dr (Intercept)	2.084	0.070	29.81	**<0.001**
Pp (Intercept)	1.793	0.104	17.33	**<0.001**
Aj HPE	0.000	0.000	−2.31	**0.02**
Cp HPE	0.004	0.002	2.38	**0.017**
Dr HPE	0.001	0.002	0.82	0.4149
Pp HPE	−0.007	0.003	−2.32	**0.02**
Aj PC1	−0.049	0.028	−1.75	0.08
Cp PC1	0.168	0.019	8.87	**<0.001**
Dr PC1	0.131	0.033	4.02	**<0.001**
Pp PC1	0.280	0.046	6.11	**<0.001**
Aj PC2	−0.141	0.035	−4.03	**<0.001**
Cp PC2	−0.266	0.028	−9.43	**<0.001**
Dr PC2	0.218	0.052	4.21	**<0.001**
Pp PC2	0.005	0.057	0.09	0.93
ZERO COMPONENT				
Ap (Intercept)	−1.637	0.385	−4.26	**<0.001**
Cp (Intercept)	−0.582	0.141	−4.12	**<0.001**
Dr (Intercept)	−1.279	0.161	−7.92	**<0.001**
Pp (Intercept)	−1.735	0.323	−5.37	**<0.001**

Real values (i.e., not relative to reference level) of the parameters are presented. Ap – *Artibeus planirostris*; Cp – *Carollia perspicillata*; Dr – *Desmodus rotundus*; Pp – *Pteronotus parnellii*. PC1 and PC2 – principal components of the seven environmental variables; HPE – Human population density estimate (see Methods for details); SE – standard error of parameter estimation. Results in bold significantly differ from zero.

The best model contained interactions between each explanatory variable and host species and the variation explained by the host species itself was high (model 6, Table 1). Therefore, we argue that the best model indicates that ectoparasite abundance is a host-specific trait. To circumvent this caveat we ran separate sets of models for each host species (Table S2). In each of these runs we assumed zero counts to have the same probability of being false zeros (Table S2), because the effect of species was controlled for. Models containing HPE had a greater model probability in *A. planirostris*, *C. perspicillata* and *P. parnellii*, as indicated by model weights (Table S2), corroborating the importance of this variable in explaining fly abundance in these species.

## Discussion

We examined the effects of anthropogenic disturbance and climate on fly abundance in four widespread species of Neotropical bats parasitized by highly host-specific bat flies. In three of four species, disturbance had a significant effect on fly abundance and climate had some effect in all four hosts. In addition, our analysis showed that fly abundance is greatly influenced by the identity of the bat species, suggesting that in the bats we examined, parasite abundance may be a host-species attribute. Individuals of some host species are consistently more parasitized than individuals of others [46]. The most likely reason behind this is among-host difference in traits that constrain the range of parasite abundance (see discussion in [47]). For example, host species may differ in levels of immunocompetence or body size. In Neotropical bats, fly abundance is not affected by body size [48], but is greatly influenced by the roosting habits [19]. Within the constraints imposed by roost use, the environment can cause variation in fly abundance by, for example, affecting fly development or the encounter rate between a bat and a fly.

The low variation explained by the explanatory variables indicated that our data was “noisy”. Nonetheless, both climate and disturbance were important factors in determining fly abundance as indicated by the model selection process. Moreover, the essence of our findings remained the same within host species when the effects of host species were eliminated by running separate analyses for each host. The best model of our more general analysis (model 1, Table 1) represents the biological hypothesis that fly abundance is affected by climate and anthropogenic disturbance (albeit differently in different bat species). Because bat flies are exposed to the environment during their life cycle, fly abundance may be directly affected by local climatic conditions, as for other ectoparasites (e.g., fleas, ticks and mites [15], [16]). It is possible, for instance, that temperature had a similar positive effect on fly abundance in three of the four host species because the development of bat flies is favored in warmer temperatures. Similarly, in *A. planirostris* and *C. perspicillata*, which roost in relatively exposed roosts, precipitation may directly affect the survival of fly pupae, which may explain the negative effect of precipitation found in these two species. Nonetheless, the climatic variables used were coarse and do not reflect the microclimate in the roosts at time of capture. Therefore, it is more plausible that our results point to indirect effects of climate on fly abundance through its impact on the host [25]. Because data on local host densities or host condition were not available, this hypothesis needs to be tested.

The effects of disturbance on parasitism can be diverse and depend on the underlying ecological processes. For example, parasite transmission is promoted where disturbance increases host contact-rates [10]. Habitat fragmentation can result in crowding of a host, thereby increasing host contact rates [4], [49]. On the other hand, the same disturbance can limit parasite transmission if it results in a decrease in host population density, for example, due to increased host mortality or longer movement bouts between patches which reduce within-host contact rate [50]. Obviously, these environmental effects interact with the life history traits of the organism. In our case, the roosting habits of the bat species should play a crucial role in mediating the effects of disturbance on fly abundance [19]. *Carollia perspicillata*, for instance, uses roost sites which are altered by human activity (e.g., trees) [28]. In the Neotropics, anthropogenic disturbance can lead to an increase in the local densities of some bats [22], [51]. An increase in the local abundance of *C. perspicillata*, a species that is strongly associated with disturbed forests [22], [52], might promote bat congregation and facilitate horizontal fly transmission. In support of this, we found a significant effect of disturbance on fly abundance.

In contrast to *C. perspicillata*, fly abundance in *A. planirostris* decreased significantly with level of disturbance, contrary to our prediction. This could be the result of a decrease in population density of *A. planirostris* in disturbed areas. Alternatively, a combination of an increase in local host abundances with a physiologically-limited fly reproduction could result in a “dilution effect” whereby fly abundance is diluted among hosts [14]. The differences in trends between *A. planirostris* and *C. perspicillata* indicate that different regulatory mechanisms could determine fly abundance in different bat species as was demonstrated for fleas parasitizing small mammals [7]. Contrary to our findings, Cottontail et al. [3] showed that the prevalence of hemoparasites in *A. planirostris* was higher in fragmented habitats than in non-fragmented ones in Panama, probably due to increased host density in the fragmented habitats. This indicates that processes that affect parasite acquisition may also vary geographically within host species [53], [54].

Results for *P. parnellii* and *D. rotundus* are more difficult to explain. The decrease in fly abundance of *P. parnellii* with disturbance may be related, at least partly, to the use of man-made roosts (e.g., buildings) by this species [28]. As these become available, the densities of bat per roost may decrease, thus lowering the rate of horizontal transmission of flies across individuals. However, in the system we investigated, most disturbed sites were not located in urban areas but rather in natural (undisturbed primary forest) or rural areas (i.e., clear-cut forests and small villages) [26] that provide little opportunity for bats to use man-made structures. Similarly to *A. planirostris* and *C. perspicillata*, *D. rotundus* also roosts in trees but fly abundance in *D. rotundus* was not affected by disturbance. This could be a result of stable population densities, or contrasting processes that operate simultaneously and result in a null effect on fly abundance.

Parasite abundance is only one measurement of parasite diversity and insight can be gained by assessing the composition of fly infracommunities, which can vary across space [55]. Although bat flies are highly host-specific, several bat fly species can parasitize a single bat host. For instance, *C. perspicillata* is parasitized by six different fly species. We calculated the total fly abundance of the infracommunity. However, it will be intriguing to examine if climate and anthropogenic disturbance alter the relative abundance of each fly species as well as the fly species composition in the infracommunity.

How host-parasite interactions are mediated by anthropogenic disturbance is intriguing. One common notion is that highly complex and context-dependent set of interactions between hosts, parasites and environment, makes it difficult to propose clear-cut predictions on how anthropogenic disturbance will affect host-parasite dynamics and associations [56]. Our findings suggest that human disturbance may play a significant role in shaping bat-bat fly interactions at least in some bat species, although the mechanisms behind the indirect effects of the environment on patterns of bat fly parasitism remain unclear. Detailed studies that consider local conditions experienced by bats and their flies are needed to characterize the underlying mechanisms that cause the observed patterns.

## Supporting Information

Figure S1 **A map of primary localities where bats were captured during the Smithsonian Venezuela Project. Numbers correspond to Handley**'**s** [**26**] **gazetteer and to Table S1.**
(TIF)

Table S1 **Mean values of the factors used in our models, calculated within a 5 km radius for each primary locality.**
(PDF)

Table S2 **Comparison of models used to test the effect of climate and disturbance on fly abundance per host species in four species of bats in the Smithsonian Venezuela Project data set.**
(PDF)

Protocol S1 **Detailed description of the zero-inflated negative binomial statistical procedures.**
(PDF)
