# Supplementary figures and images for: Effects of Anthropogenic Disturbance and Climate on Patterns of Bat Fly Parasitism
Source: PLoS One. 2012 Jul 19;7(7):e41487. doi: 10.1371/journal.pone.0041487 (PMC3400619; doi:10.1371/journal.pone.0041487)

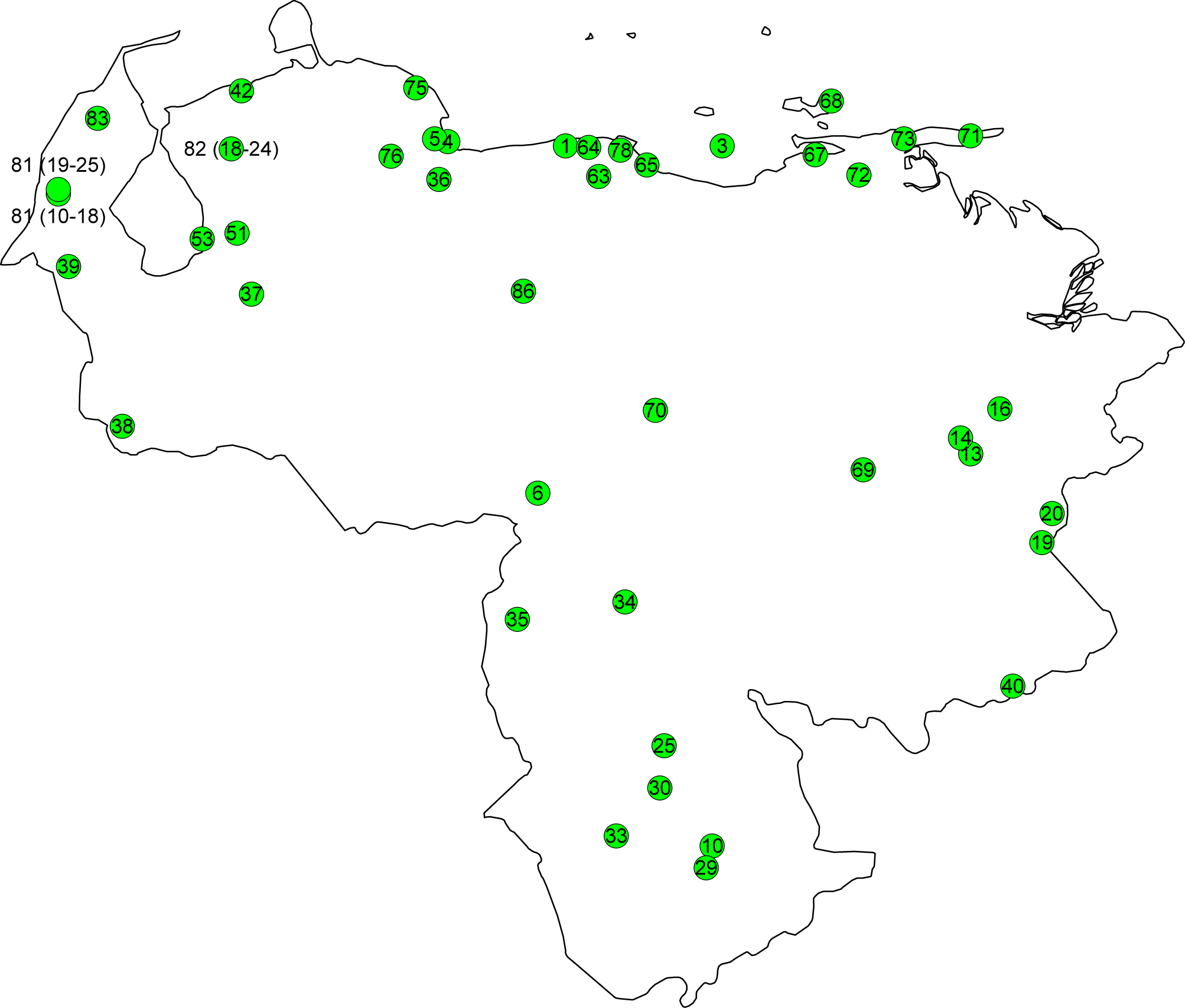

Supplement: Figure S1 — A map of primary localities where bats were captured during the Smithsonian Venezuela Project. Numbers correspond to Handley's [26] gazetteer and to Table S1. (TIF) [file pone.0041487.s001.tif]
