# Supplementary material for: Effects of Anthropogenic Disturbance and Climate on Patterns of Bat Fly Parasitism
Source: PLoS One. 2012 Jul 19;7(7):e41487. doi: 10.1371/journal.pone.0041487 (PMC3400619; doi:10.1371/journal.pone.0041487)
Supplement: Table S1 — Mean values of the factors used in our models, calculated within a 5 km radius for each primary locality. (PDF) [file pone.0041487.s002.pdf]

Table S1. Mean values of the factors used in our models.

PL	MAT (° C)	MAP (mm)	MTWQ (° C)	MTCQ (° C)	MPWQ (mm)	MPDQ (mm)	ELE (m)	HPE (people/5 km^2)
1	17.9	1020.6	18.7	16.7	395.3	98.8	1366.6	3500
10	25.9	2338.3	26.4	25.5	898.4	353.7	193.2	1
13	26	1452.7	26.1	25	653.4	145.2	229	1.3
14	25	1375.4	26	24.8	640.9	116.1	289	1
16	26	1122.5	26	25	476.9	127	202.8	2
19	19.7	1793.4	20.3	19.1	730.6	177.9	1225.7	1
20	25.9	2254.4	26	25	851.9	323	192.4	1
25	21.5	3223.7	21.9	20.9	1287.8	366	1008.6	1
29	26	2459.1	26	25	922.2	389.4	190.8	1
3	18.2	1057	18.8	16.8	433.4	92	1345.8	3500
30	26	3254.1	27	26	1295.5	418.3	125.5	1
33	26	3495	27	25.7	1294.9	514.5	111.5	1
34	26.6	2669.1	27.5	25.6	1342.5	110.1	202.6	1
35	22.5	3011.8	23.5	21.4	1391.3	183.2	952.4	1
36	22.4	1011.7	22.8	21.8	455.6	26	708.6	12
37	22.2	2110.2	22.4	21.5	867.7	125.2	1006.5	16
38	24.3	3247	24.7	23.9	1326.5	231.5	392.9	5.2
39	28	2361.9	28	27	931.3	107.4	23	1.3
4	27	1129.1	27	26	418.4	146.5	6.9	49
40	23.8	2136.3	24.2	23.6	779.4	342.3	543.2	1
42	27.8	434.2	28.7	26	235.4	12.4	31.1	2.7
5	27	1235.6	27	26	448.5	154.3	15.9	32

51	27.3	1066.6	27.7	26.7	388.1	130.1	155.5	28.3
53	28	1228.7	28	27	420.3	160.6	0.5	26
6	28	2189.2	29	26	1183.4	40.9	43.5	1
63	24	1837.2	24.7	22.8	825.6	169	404.1	33.3
64	18.9	1140.3	19.6	17.7	447	118.8	1159.8	218
65	27	1405.5	28	26	505.2	113.9	6.8	25.3
67	25.3	1051.6	25.8	24.3	471.2	70.2	179.4	162.2
68	26.4	683.3	27.2	25.2	321.5	60	123.1	125.7
69	25	1668.5	25.9	24.9	913.9	60.5	349.4	1
70	26.8	1630.3	27.8	26.6	912.9	35.2	88.2	1
71	24.6	1481	25.2	23.6	575.7	132.2	291.4	23.5
72	18.4	1381.3	18.9	17.3	512.9	172.1	1227.2	22.2
73	25.4	1397.6	26.1	24.3	621.5	126.4	173.4	44
75	25.5	1234.4	26.2	24.5	529.7	113.5	108.7	13.2
76	22.8	1101.3	23.2	21.9	435.2	70.9	619.7	14.7
78	24.4	1637.4	25.1	23.2	595.9	173.7	326.8	9.3
81(10-18)	25	2037	25.5	24.2	753.9	162.4	387.5	1.5
81(19-25)	22.8	2017.6	23.2	22	786.6	159.7	724.2	2
82(18-24)	24.1	1209.3	24.5	23.3	542.5	59.7	460.3	3.5
83	27.1	1287	28	26.2	555	41.8	87.4	14
86	27	1251.5	28	26	645.6	11.4	96.4	10

Variables were calculated within a 5 km radius for each primary locality. PL – number of primary locality as appears in Handley [1] and Fig. S1; MAT – mean annual temperature; MAP – mean annual precipitation; MTWQ – mean temperature of the warmest quarter; MTCQ – mean temperature of the coldest quarter; MPWQ –

mean precipitation of the wettest quarter; MPDQ – mean precipitation of the driest quarter; ELE – elevation; HPE – human population density estimate. Climatic variables are based on Geographic Information System layers available at WorldClim (www.worldclim.org) and on [2]. HPE is based on the Latin America and Caribbean Population Distribution Database for 1960 [3,4] provided by the United Nations Environmental Programme (http://na.unep.net/siouxfalls/datasets/datalist.php) [4].

References

1. Handley CO (1976) Mammals of the Smithsonian Venezuelan project. Brigham Young Univ Sci Bull Biol Ser 20: 1-91.

2. Hijmans RJ, Cameron SE, Parra JL, Jones PG, Jarvis A (2005) Very high resolution interpolated climate surfaces for global land areas. Int J Climatol 25: 1965-1978.

3. Tobler W, Deichmann U, Gottsegen J, Maloy K (1997) World population in a grid of spherical quadrilaterals. Int J Popul Geog 3: 203-225.

4. Hyman G (2000) Latin American population database. Eros Data Center, Sioux Falls, South Dakota, USA: UNEP/GRID-Sioux Falls.
