# Supplementary material for: Effects of Anthropogenic Disturbance and Climate on Patterns of Bat Fly Parasitism
Source: PLoS One. 2012 Jul 19;7(7):e41487. doi: 10.1371/journal.pone.0041487 (PMC3400619; doi:10.1371/journal.pone.0041487)
Supplement: Table S2 — Comparison of models used to test the effect of climate and disturbance on fly abundance per host species in four species of bats in the Smithsonian Venezuela Project data set. (PDF) [file pone.0041487.s003.pdf]

Table S2. Comparison of models used to test the effect of climate and disturbance on fly abundance per host species in four species of bats in the Smithsonian Venezuela Project data set.

Model ranks	Model structure	K	R^2	AIC	Δ AIC	w_i
Artibeus planirostris						
1 [a]	PC1+PC2+HPE	6	0.016	3386.1	0.0	98.37
2	PC2	4	0.007	3395.2	9.1	1.06
3	PC1+PC2	5	0.007	3396.8	10.6	0.48
4	HPE	4	0.002	3401.4	15.3	0.05
5 [b]	1	3	0	3402.7	16.6	0.02
6	PC1	4	0.001	3403.4	17.3	0.02
Carollia perspicillata						
1 [a]	PC1+PC2+HPE	6	0.076	7666.3	0.0	88.48
2	PC1+PC2	5	0.074	7670.4	4.1	11.52
3	PC2	4	0.041	7776.5	110.1	0
4	HPE	4	0.028	7818.3	151.9	0
5	PC1	4	0.026	7823.8	157.5	0
6 [b]	1	3	0	7902.9	236.6	0
Desmodus rotundus						
1	PC1+PC2	5	0.033	4248.7	0.0	64.96
2 [a]	PC1+PC2+HPE	6	0.034	4250.0	1.2	34.99
3	PC2	4	0.011	4263.8	15.1	0.04
4	PC1	4	0.005	4268.1	19.4	0
5 [b]	1	3	0	4270.3	21.6	0

6	HPE	4	0	4272.1	23.4	0
Pteronotus parnellii						
1 [a]	PC1+PC2+HPE	6	0.137	1830.7	0.0	92.93
2	PC1+PC2	5	0.118	1836.1	5.4	6.26
3	PC1	4	0.102	1840.2	9.5	0.81
4	PC2	4	0.011	1873.3	42.7	0
5	HPE	4	0.01	1873.7	43.0	0
6 [b]	1	3	0	1875.3	44.6	0

Models are ranked from the most supported (best model) to the least supported according to Akaike information criteria (AIC). Δ AIC – difference in AIC between the current and best model; w_i – model probabilities. K –number of parameters in the model. Note that the number of parameters includes *k*, which is the dispersion parameter of the negative binomial distribution (see Protocol S1 for details). PC1 and PC2 – principal components of the seven environmental variables; HPE – Human population density estimate (see Methods in main text for details).

[a] Global model; [b] Null model
