# Supplementary material for: Effects of Anthropogenic Disturbance and Climate on Patterns of Bat Fly Parasitism
Source: PLoS One. 2012 Jul 19;7(7):e41487. doi: 10.1371/journal.pone.0041487 (PMC3400619; doi:10.1371/journal.pone.0041487)
Supplement: Protocol S1 — Detailed description of the zero-inflated negative binomial statistical procedures. (PDF) [file pone.0041487.s004.pdf]

Protocol S1. Detailed description of the zero-inflated negative binomial statistical procedures.

In this supplemental protocol we detail the procedures used in our zero-inflated negative binomial mixture models (ZINB). We base our explanations on [1,2,3,4], where a more detailed discussion of the logic, mathematics, methodology and interpretation of ZINB models can be found. We base the examples given herein on our results.

As mentioned in the Method section in the main text, there are two sources of zeros in ecological data: "false zeros" and "true zeros" [1,2,4]. In a mixture model, the complete distribution of the estimated counts (including zeros) is represented by two separate components: a *zero component* modeling the probability of false zeros and a *count component* accounting for the true zeros and non-zero counts [1,4]. The zero component is a binomial process. Hence, the probability that observation *i* of the response variable (Y_i) is a false zero is binomially distributed with probability π_i [4]. The count component is a count process. Therefore, the probability that Y_i is a zero (false or true) equals the probability that it is a false zero plus the probability that it is not a false zero times the probability of sampling a true zero in the count process [4]:

$$P(Y_i = 0) = \pi_i + (1 - \pi_i) \cdot P(true\ zero) \qquad (1),$$

where *P* denotes probability. The count component can be modeled by a Poisson or negative binomial distribution [4]. In our models we used the negative binomial distribution as it had a better fit to the data in all cases [1]. A negative binomial distribution has a mean $E(Y) = \mu$ and variance $var(Y) = \mu + \mu^2/k$, where *k* is a dispersion parameter that determines the amount of overdispersion in the data (the smaller *k*, the larger the overdispersion) [4]. Following [1,2,4], we can take into

account the probability function of the negative binomial distribution to extend equation 1 also to a case where $Yi > 0$. We denote the probability of observation i being a zero or non-zero as:

$$\begin{cases} \mathrm{P}(Y_i = 0) = \pi_i + (1-\pi_i) \cdot \left(\dfrac{k}{\mu_i + k} \right)^k \\ \mathrm{P}(Y_i = n) = (1-\pi_i) \cdot \dfrac{\Gamma(Y_i + k)}{\Gamma(k)\Gamma(Y_i + 1)} \cdot \left(\dfrac{k}{\mu_i + k} \right)^k \cdot \left(1 - \dfrac{k}{\mu_i + k} \right)^{Y_i} \end{cases} \qquad (2)$$

where Γ is the gamma distribution and n is a natural number larger than 0. For our purposes, the details of equation 2 are of less importance. Instead, it is noteworthy that equation 2 indicates that $P(Yi)$ is a function of μ_i, π_i and k.

We can use equation 2 to incorporate covariates in our analysis. In particular, we can model π_i and μ_i as a function of a set of explanatory variables. For π_i it is common to use a logistic regression with a logit link function, as it describes a binomial process:

$$\log it(\pi_i) = e^{\alpha + \beta_1 X_1 + \beta_2 X_2 + \ldots + \beta_n X_n} \qquad (3),$$

where α is the intercept, $\beta_1 \ldots \beta_n$ are the model parameters we aim to estimate (i.e., what is presented in the zero component section of Table 2 in the main text), and $X_1 \ldots X_n$ is a set of explanatory variables such as host species or human population estimate (HPE). We can also model the dependence of μ_i on a different (or same) set of explanatory variables with the aid of a log link function:

$$\log(\mu_i) = \gamma + \delta_1 Z_1 + \delta_2 Z_2 + \ldots + \delta_n Z_n \qquad (4),$$

where γ is the intercept, $\delta_1 \ldots \delta_n$ are the model parameters we aim to estimate (i.e., what is presented in the count component section of Table 2 in the main text), and $Z_1 \ldots Z_n$ is a set of explanatory variables. The log link function ensures that the estimated μ_i will not be negative, regardless of parameter values.

Incorporating model covariates in this manner allowed us to pose our hypotheses in the familiar regression form (see Table 1 in the main text). As with any regression, continuous variables are plugged directly into the model whereas categorical variables (i.e. host species) are incorporated through the use of dummy variables to account for the different levels of the categorical factor. When a specific factor level appears in the model, its value of X or Z is set as 1, or 0 otherwise. Commonly, as well as in the software we used [2,3], when more than one factor level appears in the model, the model parameters of each dummy variable are estimated relative to a reference level chosen arbitrarily among the factor levels, while the parameter of the reference level (intercept of the model) is calculated relative to zero [5]. For instance, suppose our categorical variable is 'Species', with four levels (species). If the parameter estimate for the reference level (e.g. *Artibeus planirostris*) is -0.271 and that of *Carollia perspicillata* is 0.488, it could be inferred that the parameter estimate for *C. perspicillata* is 0.488-0.271 = 0.217 (Table 1).

For continuous variables, the slope of the linear fit of a certain level is added to that of the reference level. For example, suppose that the parameter estimate of HPE is 0.1 for *A. planirostris* (the reference level), and the parameter estimate of HPE for *C. perspicillata* is 0.2; then the true parameter estimate of HPE for *C. perspicillata* would be 0.3. We were interested in the difference of the count component parameters from *zero*. Consequently, we ran the best model four times, sequentially selecting each host species as the reference level for each run as suggested by [5].

After obtaining estimates for the parameters of the zero component of the model, we calculated the estimated odds of observing an excess zero as the exponential of the parameter estimate, following equation 3 [1]. We calculated fly abundance under a set of certain conditions (e.g., for a specific host species in a

location with specific HPE; Fig. 1) as suggested by [1, 4]. First, we used equation 3 and the parameter values we estimated for the zero component to calculate the probability π_i of a false zero. Automatically, $1-\pi_i$ is the probability of obtaining a true zero. Then, we used the parameter estimates we obtained for the count component and plugged them in equation 4 along with the appropriate explanatory variables (e.g. Species, HPE). We multiplied the result of equation 4 by $(1- \pi_i)$ to ensure each zero is a true zero.

References

1. Loeys T, Moerkerke B, Smet OD, Buysse A (2012) The analysis of zero-inflated count data: Beyond zero-inflated Poisson regression. Br J Math Stat Psychol 65: 163-180.

2. Martin TG, Wintle BA, Rhodes JR, Kuhnert PM, Field SA, et al. (2005) Zero tolerance ecology: improving ecological inference by modelling the source of zero observations. Ecol Lett 8: 1235-1246.

3. Zeileis A, Kleiber C, Jackman S (2008) Regression models for count data in R. J Stat Softw 27: 1-25.

4. Zuur AF, Ieno EN, Walker NJ, Saveliev AA, Smith GM (2009) Zero-truncated and zero-inflated models for count data. Mixed effects models and extensions in ecology With R. New York: Springer. pp. 261-293.

5. Zuur AF, Ieno EN, Smith G, M. (2007) Analysing ecological data. Berlin: Springer. 672 p.
